# Formulation of Piperine Nanoparticles: In Vitro Breast Cancer Cell Line and In Vivo Evaluation

**DOI:** 10.3390/polym14071349

**Published:** 2022-03-26

**Authors:** Imran Kazmi, Fahad A. Al-Abbasi, Syed Sarim Imam, Muhammad Afzal, Muhammad Shahid Nadeem, Hisham N. Altayb, Sultan Alshehri

**Affiliations:** 1 Department of Biochemistry, Faculty of Science, King Abdulaziz University, Jeddah 21589, Saudi Arabia; fabbasi@kau.edu.sa (F.A.A.-A.); mhalim@kau.edu.sa (M.S.N.); hdemmahom@kau.edu.sa (H.N.A.); 2 Department of Pharmaceutics, College of Pharmacy, King Saud University, Riyadh 11451, Saudi Arabia; salshehri1@ksu.edu.sa; 3 Department of Pharmacology, College of Pharmacy, Jouf University, Sakaka 72341, Saudi Arabia; afzalgufran@ju.edu.sa

**Keywords:** Box–Behnken design, chitosan, mucoadhesion, breast cancer, intestinal permeation, oral bioavailability

## Abstract

Piperine (PPN), one of the most investigated phytochemicals, is known to have excellent therapeutic efficacy against a variety of ailments including breast cancer. However, its physicochemical properties such as poor aqueous solubility restrict its clinical application. Therefore, the present investigation was designed to develop PPN encapsulated lipid polymer hybrid nanoparticles (PPN-LPHNPs) to overcome the limitation. The developed PPN-LPHNPs were optimized by the three-factor, three-level Box–Behnken design (3^3^-BBD). The optimized PPN-LPHNPs were then evaluated for their drug release profile, cytotoxicity assay against MDA-MB-231 and MCF-7 cells, and gastrointestinal stability as well as colloidal stability. In addition, the optimized PPN-LPHNPs were evaluated for ex vivo intestinal permeation and in vivo pharmacokinetic in albino Wistar rats. As per the results, the optimized PPN-LPHNPs showed a small average particles size of <160 nm with a low (<0.3) polydispersity index, and highly positive surface charge (>+20 mV). PPN-LPHNPs revealed excellent gastrointestinal as well as colloidal stability and sustained release profiles up to 24 h. Furthermore, PPN-LPHNPs revealed excellent cytotoxicity against both MDA-MB-231 and MCF-7 cancer cells compared to the free PPN. Moreover, animal studies revealed that the PPN-LPHNPs exhibited a 6.02- and 4.55-fold higher intestinal permeation and relative oral bioavailability, respectively, in comparison to the conventional PPN suspension. Thus, our developed LPHNPs present a strong potential for improved delivery of PPN.

## 1. Introduction

In the modern era of the 21st century, breast cancer (BC) still remains a major health concern in women globally. This neoplastic disease is characterized by the uncontrolled cell division of cancerous cells in the breast tissue, leading to the development of a tumor mass [1]. At present, more than 1 million women are diagnosed with BC every year [2]. Current BC treatment involves a multidisciplinary approach that involves surgery and radiotherapy as well as chemotherapy as adjuvant therapy [3]. Radiotherapy involves exposure to radiation of BC tumor and may increase the heart and lung disease due to the presence of these organs adjacent to the breast. In addition, radiotherapy increases the risk of leukemia [4]. To date, chemotherapy is the most common treatment option for BC treatment. However, chemotherapy is not a safe option for BC treatment due to the undesirable side effects. Most chemotherapeutic drugs are characterized by high lipophilicity and low water solubility and show low bioavailability and thereby low therapeutic efficacy. Moreover, chemotherapeutic drugs are unable to differentiate between the cancerous and normal healthy cells and also kill normal cells during chemotherapy. Therefore, the improvement in the solubility and site-specific targeting of the chemotherapeutic drugs is of the utmost need for the successful treatment of BC [5].

**Figure 1:**
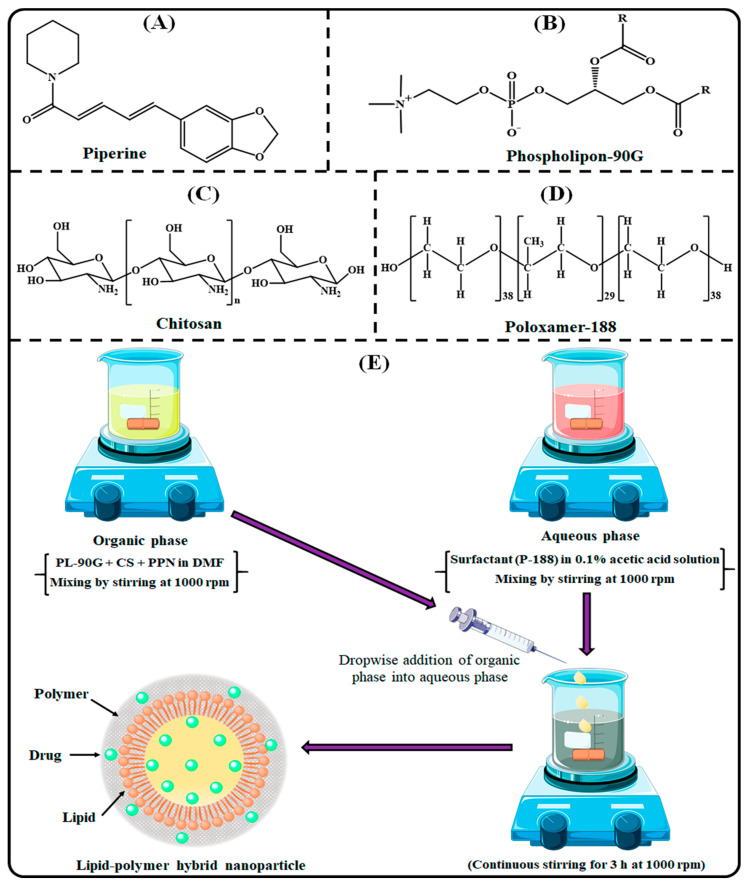
Image illustrating the chemical structure of (**A**) PPN, (**B**) PL-90G, (**C**) CS, (**D**) P-188, and (**E**) scheme for the preparation of PPN-LPHNPs.

Piperine (PPN; Figure 1A) is an alkaloid majorly obtained from the plants such as black pepper and long pepper belonging to the Piperaceae family [6]. PPN is also called the “king of spices” and has excellent medicinal properties against a variety of ailments [7]. In the era of “Naturopathy,” PPN is one of the most widely used phytochemicals to cure a wide range of ailments. In addition, PPN is considered to be the first and most potent natural bioenhancer [8]. PPN has significant potential to inhibit CYP3A4 (a metabolizing enzyme) and P-glycoprotein (P-gp; an efflux transporter) [9,10]. P-gp is basically an efflux transporter that effluxes several drugs out of the cells after absorption from the intestine [11]. Recent studies have suggested that PPN has excellent therapeutic efficacy against BC. PPN acts anti-breast cancer effect by the induction of apoptosis by arresting cell cycle, modulation of signaling protein expression, and depression in transcription factors [12]. However, the therapeutic application of PPN is very limited because of its very low overall oral bioavailability. The poor oral bioavailability of PPN corresponds to its high lipophilicity and very limited water solubility (40 μg/mL) [13]. By considering the above facts, formulation scientists across the world focus on the preparation of novel formulations based on nanotechnology to improve oral bioavailability of lipophilic compounds by increasing aqueous solubility to improve its efficacy against BC [14].

The development of nanotechnology-based formulations (i.e., nanoparticles) provides an effective way to solve the above-mentioned problem associated with chemotherapeutic drugs [15,16]. Encapsulation of chemotherapeutic drugs in the nanoparticles offers several advantages over conventional formulations. Encapsulation of bioactive compounds in the nanoparticles significantly increases the overall surface area for absorption because of small-sized particles. In addition, lipophilic drugs are encapsulated in an amorphous state in the matrix of the nanoparticles, thus improving the solubility and thereby oral bioavailability of the encapsulated drug in the nanoparticles [17]. Furthermore, the nanoparticles can deliver the encapsulated drug directly to the tumor site and can reduce undesirable side effects [18]. Among various nanocarriers, lipid–polymer hybrid nanoparticles (LPHNPs) are considered to be the best nanocarrier for the delivery of lipophilic drugs [19]. Biodegradable and biocompatible lipids, as well as polymers, are used to develop LPHNPs. LPHNPs are mainly fabricated to mitigate the challenges encountered with both lipidic as well as polymeric nanoparticles. LPHNPs exhibit combined advantages of both lipidic as well as polymeric nanocarriers [20]. A hybrid matrix of LPHNPs provides some excellent advantages over other nanoparticles such as much greater encapsulation efficiency, the tunable release of encapsulated drugs, and significantly high stability in the varying environment of the gastrointestinal tract (GIT) [21,22]. 

Generally, LPHNPs are prepared by a mixture of both biodegradable phospholipid and polymer and emulsified with surfactant. Among the phospholipids, phospholipon-90G (Figure 1B) is the most commonly used lipid to prepare nanoparticles because of its non-immunogenic as well as non-toxic properties. However, its application is limited due to instability at higher temperatures [23]. The stability of phospholipid can be increased by combining it with a suitable polymer and making an effective single system for effective drug delivery [24]. To date, chitosan (CS; Figure 1C) is the most extensively studied naturally occurring cationic, mucoadhesive, non-immunogenic, biodegradable, and biocompatible polymer and is widely used for the development of nanoparticles for oral drug delivery for the last few decades. Encapsulation of phytochemicals in the nanoparticles prepared with CS provides excellent stability in the harsh gastrointestinal (GI) pH condition and protects the phytochemicals from enzymatic degradation. In addition, due to the mucoadhesive characteristics, CS-based nanoparticles significantly increase the intestinal absorption of encapsulated drugs after oral administration [11]. Poloxamer-188 (P-188; Figure 1D) is the most common surfactant used to develop different nanocarriers due to its eco-friendly, non-ionic characteristics, and good hydrophilic–lipophilic balance [25].

By considering the above facts, we aimed to develop P-188 emulsified PL-90G and CS-based PPN encapsulated LPHNPs for enhanced oral efficacy against breast cancer. The formulation was optimized by the Box–Behnken design (BBD), and the potential of the optimized formulation was evaluated for its enhanced cytotoxicity against different BC cells and enhancement in the oral bioavailability of the PPN.

## 2. Materials and Methods

### 2.1. Materials

Piperine (PPN), Chitosan (CS; Polymer; 85% deacetylation), dialysis tube (MWCO: 12 kDa), and type II Mucin protein from the porcine stomach was purchased from Sigma-Aldrich, St. Louis, MO, USA. Phospholipon 90G (Lipid; PL-90G) was duly gifted by Lipoid, GmbH, Germany. Poloxamer-188 (P-188; Surfactant) was kindly gifted by BASF, Mumbai, India. MDA-MB-231 and MCF-7 breast tumor cells were procured from National Centre for Cell Science (NCCS), Maharashtra, India. All other chemicals with high purity were purchased from Merck India (Mumbai, India). Albino Wistar rats (220–240 g weight) of either sex were used for experiments. The animals were housed in standard conditions and fed with a pellet diet (Lipton, Mumbai, India) and water ad libitum. 

### 2.2. Experimental Design

For the development of PPN-LPHNPs, the ratio of excipients was optimized by a 3-factor, 3-level Box–Behnken design (3^3^-BBD) by using Design-Expert^®^ software V.11.0. For the experimental design, the concentration of PL-90G (coded A; 75–125 mg), the concentration of CS (coded as B; 40–80 mg), and concentration of P-188 (coded as C; 50–100 mg) was selected as independent factors/variables at 3 different levels of high (coded as “+1”) medium (coded as “0”), and low (coded as “−1”), respectively, as represented in Table 1. On the other hand, the particle size (PS; coded as R_1_), polydispersity index (PDI; coded as R_2_), and %entrapment efficiency (%EE; coded as R_3_) of the PPN-LPHNPs were considered to be the dependent factors/responses. After running the fitted data in 3^3^-BBD, 15 compositions of excipients were obtained for the optimization of PPN-LPHNPs. After that, all 15 PPN-LPHNPs were developed according to the composition and the actual values were put in the design as summarized in Table 2. Then, different statistical models such as linear, 2-F1, and quadratic were analyzed for the selection of the best-fitting model by one-way analysis of variance (ANOVA). The selected model was further explained by a polynomial equation and different plots produced from the software.

### 2.3. Development of PPN-LPHNPs

PPN-LPHNPs were developed as per the previously reported nanoprecipitation technique with a minor modification [26,27], and a scheme for the preparation is illustrated in Figure 1E. Briefly, an aqueous phase was prepared by dissolving CS (40–80 mg) in 0.1% acetic acid solution. Then, an accurately weighed quantity of P-188 (50–100 mg) was added and dissolved properly by gentle agitation. Separately, the lipid phase was prepared by adding an accurately weighed amount of PL-90G (75–125 mg) and PPN (20 mg) in 2 mL of N,N-dimethylformamide by continuous stirring with a magnetic stirrer. After that, the lipid phase was added dropwise with a 1 mL syringe to the aqueous phase at a constant flow rate under a constant stirring speed of 1000 rpm. The resulting mixture was stirred for 3 h for the self-assembling of the PPN-LPHNPs at room temperature.

### 2.4. Characterization of PPN-LPHNPs

#### 2.4.1. Particles Characterization

The prepared PPN-LPHNPs were evaluated for PS, PDI, and zeta potential (ZP) by using zeta sizer (Malvern, ZS 900, Malvern, UK). Just before the experiment, 0.3 mL of PPN-LPHNPs was diluted up to 3 mL with deionized water. The measurement was carried out at 25 °C temperature and the scattering angle was set to 90°. The morphology of the optimized PPN-LPHNPs was observed under a scanning electron microscope (JSM 6360A, JOEL, Tokyo, Japan). The samples were coated with gold and visualized under the microscope.

#### 2.4.2. Entrapment Efficiency (%EE) and Loading Capacity (%LC)

The %EE and %LC of PPN-LPHNPs were quantified by the indirect method as reported by Thakur et al. [28]. Briefly, PPN-LPHNPs were centrifuged for 30 min at 14,000 rpm with the help of a cooling centrifuge (Sigma 3K30, Sigma Laboratory, Osterode am Harz, Germany). After that, the supernatant was taken and filtered through a 0.2 µm pore-sized membrane filter. Then, the unentrapped PPN was measured with the help of UV-Spectrophotometer (Shimadzu, UV-1601 model, Kyoto, Japan) at 342 nm. The %EE and %LC were calculated using the following equations:

(1) $$\%\mathrm{EE}\;=\frac{\mathrm{Total}\;\mathrm{PPN}\;-\;\mathrm{Unencapsulated}\;\mathrm{PPN}\;}{\mathrm{Total}\;\mathrm{PPN}}\times 100$$

(2) $$\%\mathrm{LC}\;=\frac{\mathrm{Total}\;\mathrm{PPN}\;-\;\mathrm{Unencapsulated}\;\mathrm{PPN}}{\mathrm{Weight}\;\mathrm{of}\;\mathrm{LPHNPs}}\times 100$$

### 2.5. Stability Studies

#### 2.5.1. Gastrointestinal Stability

Excellent stability of PPN-LPHNPs in the GI milieu is an important factor for successful oral delivery. The stability of PPN-LPHNPs was examined in simulated gastric fluids (SGF; pH 1.2) for 2 h as well as in simulated intestinal fluids (SIF; pH 6.8) for 6 h [29]. For conducting the experiment, 2 mL of PPN-LPHNPs was poured in 10 mL of SGF and SIF, mixed properly, and incubated for predetermined time intervals. After the incubation period (2 h for SGF and 6 h for SIF), the samples were taken and the PS, PDI, %EE, and ZP were measured.

#### 2.5.2. Colloidal Stability

This study was performed to investigate the change in the PS, PDI, and %EE of PPN-LPHNPs during different storage conditions [30]. The colloidal stability of PPN-LPHNPs was examined for 180 days as per the ICH guidelines in 3 different temperature conditions (i.e., 4 ± 1 °C, 25 ± 2 °C, and 40 ± 2 °C). A measure of 5 mL of PPN-LPHNPs was taken and properly sealed in a glass vial and stored in a stability chamber (KBF-240 model, Binder Gmb, Tuttlingen, Germany). After that, the PS, PDI, and % EE were measured after pre-decided time points, i.e., 30th, 60th, 90th, 120th, 150th, and 180th days.

### 2.6. PPN Release Study

The release of PPN from the optimized PPN-LPHNPs and PPN suspension was conducted by the dialysis bag (MWCO: 12 kDa) method [31]. In this study, a 500 mL volume of SIF containing Tween 80 (0.5% *v/v*) was used as the dissolution media. An aliquot of 2.5 mL of PPN-LPHNPs was transferred in the dialysis bag and plunged in the release media at 37 °C under the constant stirring speed of 100 rpm. At pre-decided time points, 2 mL of samples were withdrawn and replaced with an equal (i.e., 2 mL) volume of fresh release media to maintain the volume of release medium. After that, the withdrawn samples were diluted and the released quantity of PPN at that time point was quantified with the help of a UV spectrophotometer at 342 nm. Drug release from PPN suspension was estimated by the same procedure. Furthermore, the obtained results were fit to different mathematical models such as zero order, first order, Higuchi, and Korsermeyer–Peppas model to understand the mechanism of PPN release as per reported method [27].

### 2.7. Bioadhesion Study

The mucoadhesive strength of the optimized PPN-LPHNPs and PPN suspension was evaluated by using mucin-type II from porcine [32]. The mucin solution (0.5% *w/v*) was prepared in phosphate buffer (pH 5.5) and incubated with optimized PPN-LPHNPs and PPN suspension (1:1) at 37 ± 1 °C for 2 h with continuous shaking (IKA-Werke, Staufen, Germany). After that, the samples were centrifuged for 30 min at 10,000 and the supernatant was collected. Finally, free mucin content was analyzed with a UV spectrophotometer at 238 nm [33].

### 2.8. Cytotoxicity Study

The breast cancer efficacy of the optimized PPN-LPHNPs and free PPN was evaluated against MDA-MB-231 and MCF-7 cells by methyl-thiazolyl-tetrazolium (MTT) colorimetric assay [34]. Firstly, breast cancer cells were seeded with 1 × 10^5^ cells per well in 96-well plates and subjected to incubation for 24 h. After confluence, cancer cells were treated with the optimized PPN-LPHNPs, free PPN, and blank LPHNPs at different concentrations and incubated for different periods (i.e., for 48 h and 72 h). After a pre-decided time (i.e., 48 h and 72 h), 25 μL of MTT dye (prepared in phosphate buffer saline; 0.5 mg/L concentration) was added to each well and plate, and then all the plates were again incubated for 3 h to develop formazan crystals. After incubation, excess culture media was removed, and formazan crystals were solubilized in 100 μL DMSO. Then, plates were gently shaken on a shaker for complete solubilization. After that, the optical density of soluble formazan was measured by a microplate reader (Bio-Rad, Hercules, CA, USA) at a wavelength of 570 nm. Finally, the IC_50_, i.e., half of the maximum inhibitory concentrations of the optimized PPN-LPHNPs and free PPN, was estimated with the help of GraphPad Prism software, 7th version for both cell lines at each time point.

### 2.9. Ex Vivo PPN Permeation Study

The potential of PPN-LPHNPs and PPN suspension permeation was evaluated in the intestine of albino Wistar rats as per the reported procedure [35]. The albino rats (202–240 g) were fasted overnight and sacrificed the next day by cervical dislocation. After that, the intestine was taken and a 5 cm-long section was sliced and washed at leashed three times to remove any food residues from the Tyrode solution. After that, PPN-LPHNPs and PPN-suspension (~2 mg of PPN) were poured into the intestinal gut sac and both ends were ligated with thread. Then, the intestine filled with the samples was immersed in 500 mL release media (i.e., Tyrod solution) at 37 ± 2 °C for 3 h, continuously stirred at 50 rpm. During the experiment, the samples were continuously aerated by an aerator (95%, O_2_). The aliquots (5 mL) were taken at pre-decided time points (i.e., just after 15, 30, 45, 60, 75, 90, 105, 120, 135, 150, 165, and 180 min) and replaced with an equal volume of fresh release media to maintain the volume of the medium. In the end, the concentration of permeated PPN from the samples was measured as per the reported RP-HPLC method [36]. For RP-HPLC analysis, a C_18_ column (250 ± 4.6 mm, Eurospher 100 with 5 μm) was used for chromatographic separation of PPN. The mobile phase was a mixture of acetonitrile and water in a ratio of 60:40. The sample was filtered by a 0.22 µm filter, and the injection volume was 20 µL. In the end, the flux was calculated from the obtained data, and the apparent permeability coefficient (APC) as well as enhancement ratio (ER) were determined by the given equation:

(3) $$\mathrm{APC}\;=\frac{\mathrm{Flux}}{\mathrm{Area}\;\mathrm{of}\;\mathrm{Sac}\;\times \mathrm{Total}\;\mathrm{quantity}\;\mathrm{of}\;\mathrm{PPN}}$$

(4) $$\mathrm{ER}\;=\frac{\mathrm{APC}\;\mathrm{of}\;\mathrm{PPN}-\mathrm{LPHNPs}}{\mathrm{APC}\;\mathrm{of}\;\mathrm{PPN}-\mathrm{Suspension}}$$

### 2.10. Pharmacokinetic Study

To evaluate the potential of LPHNPs to enhance the bioavailability of PPN compared to the conventional PPN suspension, the pharmacokinetic study was performed in albino Wistar rats. To conduct this experiment, rats were fasted overnight and then divided into two groups with six rats in each group (n = 6). Group I was fed with conventional PPN suspension and group II was administered the optimized PPN-LPHNPs. The dose of PPN was fixed to 20 mg/kg as per the reported protocol [33]. A single dose of PPN-LPHNPs and PPN suspension (1 mL, ~20 mg/kg) was fed orally to rats with the help of an oral feeding needle. At each pre-decided time point (i.e., just after 0.5, 1, 2, 4, 6, 8, 12, 24, and 48 h), 0.2 mL of blood was taken in the EDTA-coated tubes from the retro-orbital plexus. The tube containing the blood was centrifuged at 8000 rpm for 10 min to separate the plasma. After that, the PPN was extracted by 1 mL of acetone, vortexed for 10 min and subjected to centrifugation at 14,000 rpm for 5 min. The supernatant was removed and diluted properly with the mobile phase. Then, the samples were evaluated in the plasma at each time point by RP-HPLC [36]. Finally, different pharmacokinetic parameters were calculated from the plasma PPN concentration and time profiles.

### 2.11. Statistical Analysis

All the experiments are performed at least three times; the data are shown as mean ± standard deviation (SD). The results were analyzed statistically by one-way ANOVA followed by Student’s *t*-test. When the *p*-value was below 0.05, the data were considered significant. The analysis was conducted utilizing GraphPad Prism version 7.

## 3. Results and Discussion

### 3.1. Optimization of PPN-LPHNPs by Statistical Design

As per the composition suggested by the 3^3^-BBD, all the 15 PPN-LPHNPs were developed and their values were fitted in the design to select the optimized composition (Table 2). The selected responses, i.e., PS (coded as R_1_), PDI (coded as R_2_), and %EE (coded as R_3_), were fitted in the design and analyzed using different statistical models, i.e., linear, two-factor interaction (2FI), and quadratic by linear regression analysis, to select the best-fitting model. The statistical model that represents the highest (closest to 1) adjusted as well as predicted R^2^ was chosen as the best-fitting model (Table 3). Furthermore, significant terms of the best-fitting model on each response were analyzed by ANOVA, and the results are summarized in Table 4. When the terms were less than 0.05 (i.e., *p* < 0.05), the model was considered significant.

From the obtained results as summarized in Table 3, the quadratic model showed the highest adjusted as well as predicted R^2^ for all three responses. The lack-of-fit value for the quadratic model for each response was not significant (*p* < 0.05), and the variation between the actual and predicted value was very low. The statistical plots, i.e., 3D surface, contour, perturbation, and predicted vs. actual plots generated from the design, were utilized to investigate the influence of independent factors (i.e., A, B, and C) on responses (i.e., R_1_, R_2_, and R_3_). In addition, the polynomial equations obtained from the design for each response were further utilized to investigate the effect of independent factors on dependent factors.

#### 3.1.1. Effect on R_1_ (PS)

The PS of PPN-LPHNPs was observed from 27.47 nm to 218.76 nm as reported in Table 2. From the results, it can be inferred that the PS of PPN-LPHNPs was small (<225 nm) enough for effective oral delivery. The ANOVA analysis of the quadratic model suggested that all the three independent factors have a significant (*p* < 0.0001) effect on R_1_ (PS). The polynomial equation for R_1_ (PS) generated from Design Expert^®^ software is as follows:

R_1_ (PS) = +159.59 + 23.24A + 21.80B − 18.16C + 0.2475AB − 11.91AC + 0.525BC + 0.6133A^2^ + 12.66B^2^ + 0.5408C^2^ (5)

**Figure 2:**
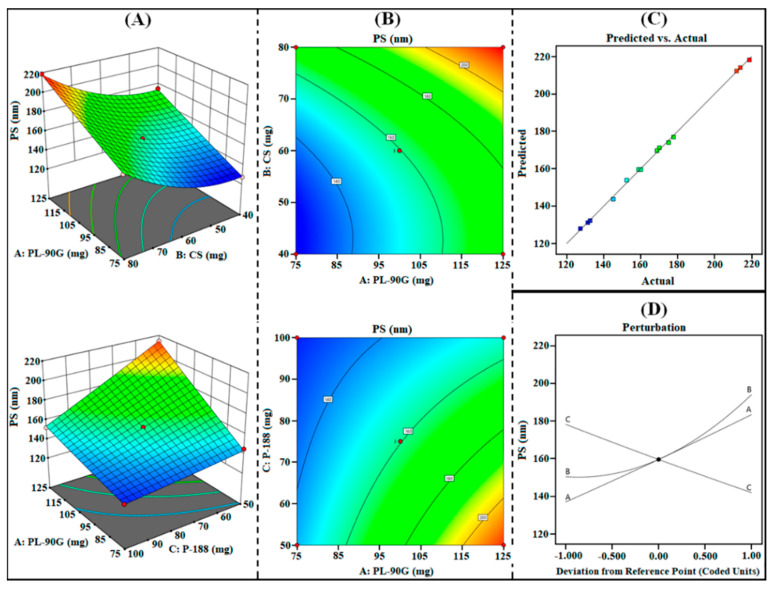
Image representing 3D response surface plots (**A**), contour plots (**B**), predicted vs. actual plot (**C**), and perturbation plot (**D**) representing the influence of independent factors (A = PL-90G; B = CS; C = P-188) on R_1_ (PS) for PPN-LPHNPs.

The statistical plots, i.e., 3D surface, contour, perturbation, and predicted vs. actual plots as shown in Figure 2A–D and polynomial Equation (5) suggested that the increase in the concentration of all independent factors from low (“–1”) to medium (“0”) and then to high (“+1”) significantly influence the PS of PPN-LPHNPs. As per the results, PS significantly increases by increasing the amount of PL-90G (coded as “A”) and CS (coded as “B”). An increment in the PL-90G concentration from 75 mg to 125 mg significantly increases the viscosity of organic solution during the preparation and greatly increases the interfacial tension between organic and aqueous phases that led to the coalescence of PL-90G, which produce LPHNPs with high PS [30]. Similarly, an increment in the CS concentration from 40 mg to 80 mg significantly increases the viscosity of the organic phase, which leads to the development of larger aggregates that ultimately produce LPHNPs with high PS [28,37]. Moreover, the PS of PPN-LPHNPs significantly reduces on increasing the amount of P-188 (coded as “C”). An increment in the P-188 concentration from 50 mg to 100 mg significantly reduces the interfacial tension and enhances the emulsification of the organic phase with the aqueous surfactant solution that resulted in the development of small-sized PPN-LPHNPs [38].

#### 3.1.2. Effect on R_2_ (PDI)

The PDI of PPN-LPHNPs was observed from 0.146 to 0.436, as reported in Table 2. From the results, it can be inferred that there was the excellent uniformity between the PS of PPN-LPHNPs. The ANOVA analysis of the quadratic model suggested that all the three independent factors have a significant (*p* < 0.0001) effect on R_2_ (PDI). The polynomial equation for R_2_ (PDI) generated from Design Expert^®^ software is as follows:

R_2_ (PDI) = +0.2313 + 0.0694A + 0.0532B − 0.0424C + 0.0385AB − 0.0077AC − 0.0075BC + 0.0120A^2^ + 0.0377B^2^ + 0.0095C^2^ (6)

**Figure 3:**
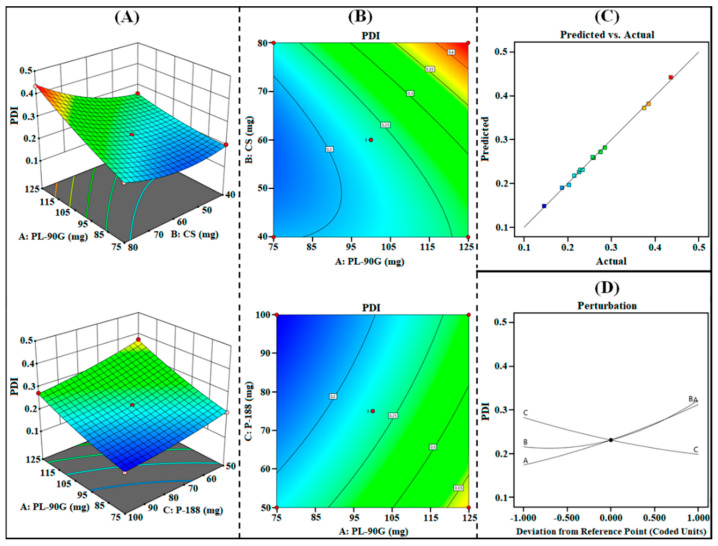
Image representing 3D response surface plots (**A**), contour plots (**B**), predicted vs. actual plot (**C**), and perturbation plot (**D**) representing the influence of independent factors (A = PL-90G; B = CS; C = P-188) on R_2_ (PDI) for PPN-LPHNPs.

The statistical plots, i.e., 3D surface, contour, perturbation, and predicted vs. actual plots as shown in Figure 3A–D and polynomial Equation (6), suggest that the increase in the concentration of all independent factors from low (“–1”) to medium (“0”) and then to high (“+1”) significantly influence the PDI of PPN-LPHNPs. As per the results, PDI significantly increases by increasing the amount of PL-90G (coded as “A”) and CS (coded as “B”). An increment in the PL-90G concentration from 75 mg to 125 mg significantly increases the viscosity of organic solution during the preparation that significantly increases the heterogeneity among the PPN-LPHNPs, which produce LPHNPs with high PDI [39]. Furthermore, an increment in the CS concentration from 40 mg to 80 mg supports the formation of coarse dispersion during the preparation due to lack of energy to combat the viscous forces and produce PPN-LPHNPs with high PDI [40]. Moreover, the PDI of PPN-LPHNPs significantly reduces on increasing the amount of P-188 (coded as “C”). An increment in the P-188 concentration from 50 mg to 100 mg significantly reduces the interfacial tension and enhances the emulsification of the organic phase with the aqueous surfactant solution, which leads to the development of small-sized PPN-LPHNPs with excellent homogeneity [27].

#### 3.1.3. Effect on R_3_ (EE)

The %EE of PPN-LPHNPs was observed from 57.98% to 81.34% as reported in Table 2. The ANOVA analysis of the quadratic model suggested that all the three independent factors have a significant (*p* < 0.0001) effect on R_3_ (%EE). The polynomial equation for R_3_ (%EE) generated from Design Expert^®^ software is as follows:

R_3_ (%EE) = +78.31 + 5.45A + 6.24B + 1.20C − 0.9825AB −2.69AC + 1.91BC − 3.73A^2^ − 3.94B^2^ − 5.56C^2^ (7)

**Figure 4:**
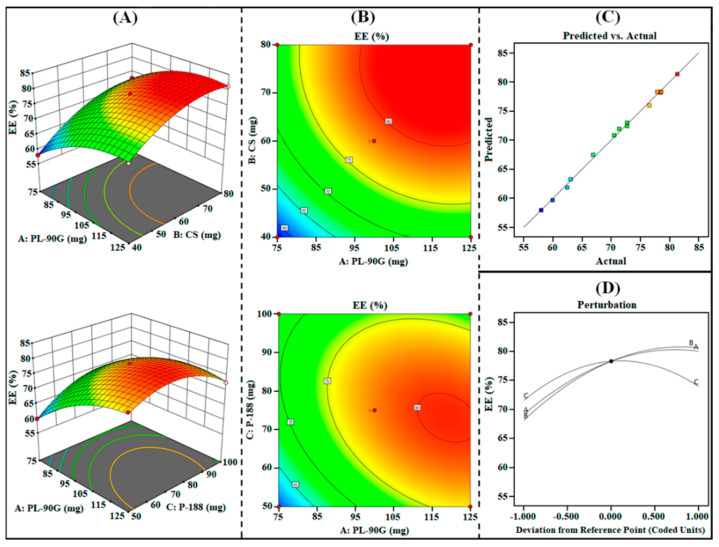
Image representing 3D response surface plots (**A**), contour plots (**B**), predicted vs. actual plot (**C**), and perturbation plot (**D**) representing the influence of independent factors (A = PL-90G; B = CS; C = P-188) on R_3_ (%EE) for PPN-LPHNPs.

The statistical plots, i.e., 3D surface, contour, perturbation, and predicted vs. actual plots as shown in Figure 4A–D and polynomial Equation (7), suggested that the increase in the concentration of all independent factors from low (“–1”) to medium (“0”) and then to high (“+1”) significantly influences the %EE of PPN-LPHNPs. As per the results, %EE significantly increases by increasing the amount of PL-90G (coded as “A”), CS (coded as “B”) as well as P-188 (coded as “C”). An increment in the PL-90G concentration from 75 mg to 125 mg significantly increases the viscosity of the organic phase, leading to rapid solidification. Rapid solidification of PL-90G significantly restricts the diffusion of PPN from the external phase, which, in turn, produces LPHNPs with high %EE [41]. Furthermore, an increment in the CS concentration from 40 mg to 80 mg significantly increases the space for entrapment of PPN and produces a compact matrix that provides PPN-LPHNPs with a high %EE [42]. In addition, the %EE of PPN-LPHNPs significantly increases on increasing the P-188 concentration from 50 mg to 75 mg. An increment in the P-188 concentration provides higher emulsification of lipid phase with surfactant solution and produces PPN-LPHNPs with high %EE. However, increment in P-188 concentration from 75 mg to 100 mg resulted in the reduction in the PS of PPN-LPHNPs. The reduction in the %EE on increasing the surfactant concentration after a certain limit ascribed to the enhancement in the partitioning of PPN from the internal to external phase results in the development of PPN-LPHNPs with low %EE [43].

#### 3.1.4. Optimized PPN-LPHNPs 

Among the 15 compositions obtained from the 3^3^-BBD that fulfill the criteria by “trading off”, all responses with a small PS, low PDI, and high %EE were chosen as the optimized composition. The point prediction method was used to select the optimized composition of PPN-LPHNPs prepared with 95 mg of PL-90G (lipid), 45 mg of CS (polymer), and 87 mg P-188 (surfactant). It showed the PS of 151.2 ± 4.12 nm, PDI of 0.213 ± 0.01, and %EE of 83.54 ± 2.88%. The software showed the predicted PS of 156.1 nm, PDI of 0.209, and %EE of 85.12%. It showed the overall desirability value of 0.939. The percent error was calculated, and it showed the value of −3.13%, 1.9%, −1.8%.

### 3.2. Characterization of PPN-LPHNPs

#### 3.2.1. Particles Characterization

**Figure 5:**
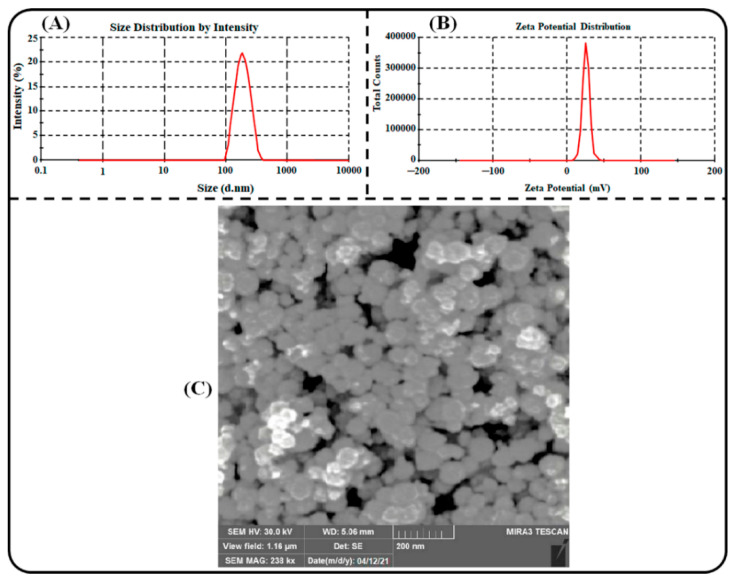
Image representing (**A**) particle size and particle size distribution, (**B**) zeta potential distribution, and (**C**) SEM micrograph of the optimized PPN-LPHNPs.

For ideal oral delivery, the size of nanoparticles should be small to attain the maximum surface for higher intestinal absorption and thereby oral bioavailability. In our investigation, the optimized PPN-LPHNPs exhibited an average PS of 151.2 ± 4.12 nm, as represented in Figure 5A. The PDI plays a significant role in the development of excellent nanoparticles for effective delivery. The PDI should be low because it indicates the uniformity among the nanoparticles in a system. The PDI value <0.3 for polymeric nanocarrier represents good homogeneity [44]. In our investigation, the optimized PPN-LPHNPs exhibited an average PDI of 0.213 ± 0.02. ZP plays a significant role in the colloidal stability of LPHNPs. A high ZP vale (>20 mV) is considered as an ideal surface charge for colloidal nanoparticles because a high surface charge repels each other, and the chance of aggregation becomes negligible and shows excellent colloidal stability [45]. In our investigation, the optimized PPN-LPHNPs exhibited an average ZP of +24.31 ± 2.41 mV, as depicted in Figure 5B. A positive value of ZP was observed due to the presence of CS in the outer shell and on the surface of PPN-LPHNPs. A positive ZP is advantageous for the oral delivery of phytochemicals because it adheres to the negatively charged mucosa of the intestinal membrane, produces significantly greater residence time in the GIT, and improves the intestinal absorption and thereby bioavailability of phytochemicals [46]. An SEM micrograph of the PPN-LPHNPs is depicted in Figure 5C. The shape of the optimized PPN-LPHNPs was spherical and uniformly distributed as well as separated from each other.

#### 3.2.2. %EE and %LC

For the successful development of LPHNPs, the %EE and %LC should be optimum for effective oral delivery. In our investigation, the optimized PPN-LPHNPs revealed an %EE and %LC of 83.54 ± 2.88%, and 6.71 ± 0.31%, respectively. Overall, an optimum and acceptable %EE and %LC was obtained for PPN-LPHNPs because of the hybrid matrix.

### 3.3. Stability Studies

#### 3.3.1. Gastrointestinal Stability

LPHNPs were prepared for the oral administration of PPN; our goal was to maintain the physicochemical parameters of PPN-LPHNPs in SGF as well as in SIF by protecting them from acidic/or enzymatic degradation during passage through the GIT. The results of the present study are summarized in Table 5. According to the results, our developed PPN-LPHNPs were stable in both SGF as well as in SIF because it revealed insignificant (*p* > 0.05) changes in their physicochemical parameters. After the incubation of PPN-LPHNPs for 2 in SGF, the PS, PDI, %EE, and ZP were observed to be 174.26 ± 5.71 nm, 0.253 ± 0.024, 74.37 ± 4.17%, and +21.07 ± 2.14 mV, respectively. After the incubation of PPN-LPHNPs for 6 h in SIF, the PS, PDI, %EE, and ZP were observed to be 169.67 ± 4.57 nm, 0.235 ± 0.017, 72.63 ± 3.54%, and +22.72 ± 2.36 mV, respectively. As per the results, it was concluded that the optimized PPN-LPHNPs were stable in gastrointestinal fluids.

#### 3.3.2. Colloidal Stability

**Figure 6:**
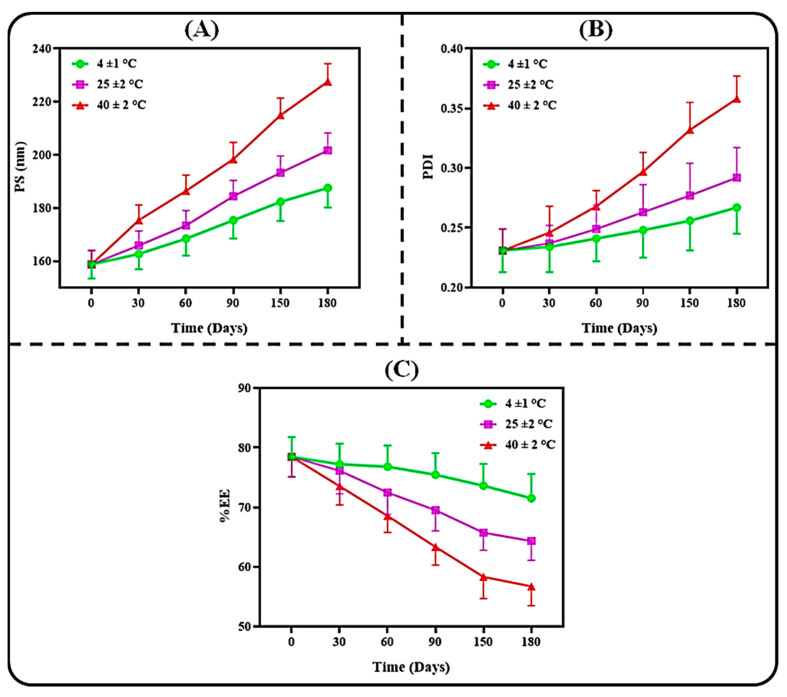
Colloidal stability of PPN-LPHNPs at different environmental conditions and different time intervals on (**A**) PS, (**B**) PDI, and (**C**) %EE.

Since the environmental condition significantly affects the stability of pharmaceutical preparations during storage, this study was carried out to understand the ability of our developed PPN-LPHNPs to maintain their integrity in different temperature conditions. The results of the present study are depicted in Figure 6. As per the results, our developed PPN-LPHNPs exhibited excellent stability in 4 ± 1 °C, even after 6 months of the study, with only insignificant changes (*p* > 0.05) in the physicochemical parameters, i.e., PS, PDI, and %EE. Similarly, at a 25 ± 2 °C storage temperature, our developed PPN-LPHNPs also exhibited insignificant (*p* > 0.05) changes in PS and PDI, as well as in %EE after 6 months of study, and revealed acceptable stability at room temperature. However, at a 40 ± 2 °C storage temperature, significant (*p* < 0.05) changes were observed in the PS and PDI, as well as in %EE of PPN-LPHNPs, suggesting poor stability. A significant (*p* < 0.05) variation in these physicochemical parameters at 40 ± 2 °C was ascribed to the conglomeration of PPN-LPHNPs because of the degradation of the polymer at high temperatures [47]. Therefore, this study suggested that our developed PPN-LPHNPs should be stored at <25 °C to maintain their integrity as well as physicochemical parameters.

### 3.4. PPN Release Study

**Figure 7:**
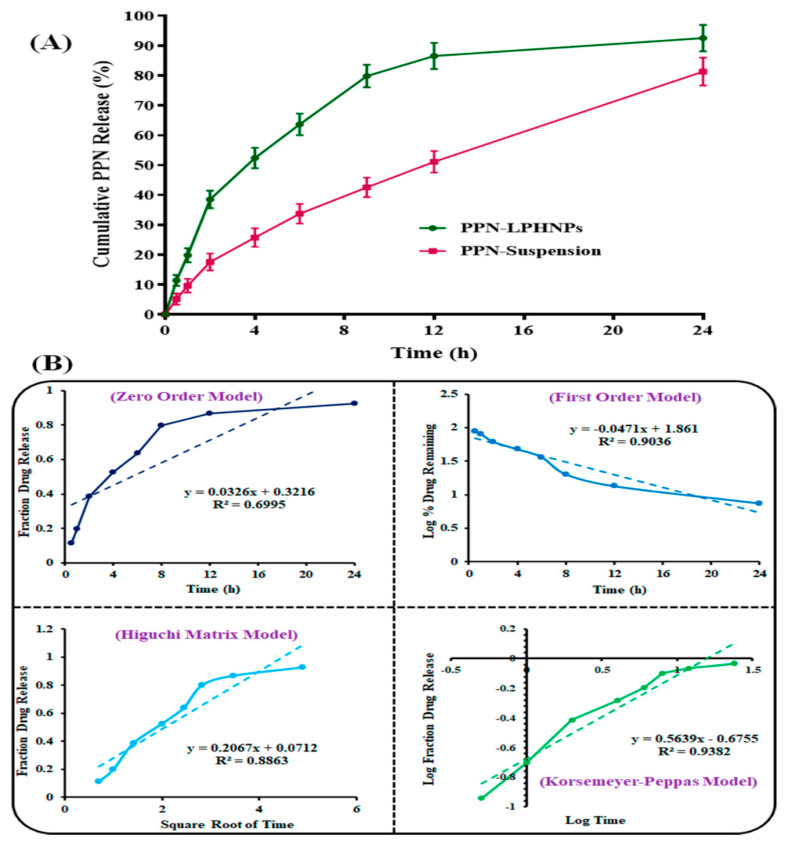
Image representing (**A**) PPN release profiles from the optimized PPN-LPHNPs and PPN suspension and (**B**) various kinetic models for optimized PPN-LPHNPs to understand the mechanism.

Figure 7A represents the release profiles of PPN-LPHNPs and PPN suspension. As per the result, the optimized PPN-LPHNPs exhibited an initially fast cumulative release of 38.54 ± 3.12% PPN after 2 h of study. In the first 2 h of the study, a fast PPN release was observed due to the dissolution of PPN from the surface of LPHNPs as well as a fast diffusion of the drug from the outer shells of LPHNPs’ hybrid matrix. From 2 to 24 h, the optimized PPN-LPHNPs showed a sustained release of PPN from LPHNPs. After 24 h of study, the total cumulative PPN release from LPHNPs was observed to be 93.56 ± 5.11%. Controlled release of PPN from LPHNPs between 2 h to 24 h was ascribed to the release of PPN from the lipophilic inner shells of the hybrid solid matrix of LPHNPs composed of PL-90G and CS [28]. On the other hand, the PPN suspension revealed 81.34 ± 4.67% release in 24 h. The lesser release of PPN from conventional suspension compared to the LPHNPs was ascribed to the poor solubility of free PPN in SIF (pH 6.8). A highly significant (*p* < 0.05) improvement in the release observed at 12 h from the optimized LPHNPs was attributed to the encapsulation of PPN in the hybrid matrix of LPHNPs in the amorphous state.

To analyze the kinetics of PPN release from LPHNPs, the release data of the optimized PPN-LPHNPs were fitted in various release kinetic models, and the obtained outputs are represented in Figure 7B. As per Figure 7B, the Korsmeyer–Peppas model revealed the maximum R^2^ (closest to 1) with R^2^ = 0.9423. Hence, the Korsmeyer–Peppas model was selected to be the best-fitting model, and the release exponent (“n”) from this model was calculated. The “n” value from the model was observed to be 0.244 and was less than 0.5. Therefore, it was suggested that the mechanism of PPN release from the LPHNPs was the “Fickian diffusion”. Fickian diffusion represents that our optimized formulation releases the encapsulated drug as per Fick’s law. Fick’s law is based on the time and concentration-dependent diffusion-controlled flow of solutes [48]. Our findings corroborated with a previous finding [27].

### 3.5. Bioadhesion Study

In the present investigation, the mucoadhesive efficiency of PPN-LPHNPs and PPN suspension was found to be 87.56 ± 6.63% and 18.37 ± 2.15%, respectively. A significant (*p* < 0.05) improvement in the mucoadhesive characteristic with PPN-LPHNPs was observed due to the presence of CS in the outer core of LPHNPs. Positive surface-charged LPHNPs strongly bind to the negatively charged mucin by electrostatic interaction that resulted in significantly higher mucoadhesion [49]. Mucoadhesive PPN-LPHNPs reside on the intestinal mucosa for a longer time, which led to higher absorption of encapsulated drugs after oral administration.

### 3.6. Cytotoxicity Study

**Figure 8:**
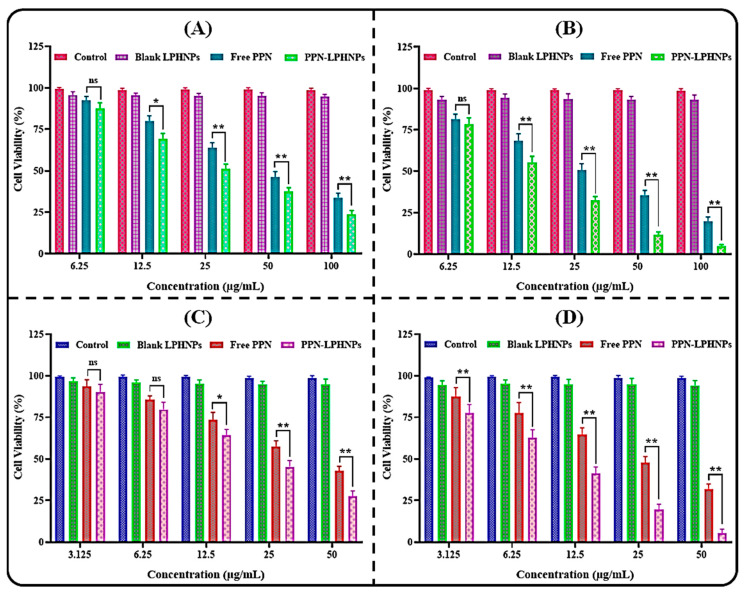
Image representing cytotoxicity assay of free PPN and PPN-LPHNPs against (**A**) MDA-MB-231 cells after 48 h, (**B**) MDA-MB-231 cells after 72 h, (**C**) MCF-7 cells after 48 h, and (**D**) MCF-7 cells after 72 h. Results are represented as a percent mean ± SD (n = 3). ns, * and ** represent non-significant, significant and highly significant differences between the two groups.

The comparative cytotoxic potential of the optimized PPN-LPHNPs and free PPN against MDA-MB-231 and MCF-7 breast cancer cells at 48 and 72 is represented in Figure 8. In this study, the optimized PPN-LPHNPs and free PPN revealed concentration- as well as time-dependent cytotoxicity against both breast cancer cells. At almost every concentration and time point (i.e., 48 as well as 72 h), PPN-LPHNPs represented significantly (*p* < 0.05) greater cytotoxicity in comparison to the free PPN against both cancer cells. After 48 h of incubation, the IC_50_ of the optimized PPN-LPHNPs and free PPN was observed to be 27.48 ± 3.37 µg/mL and 43.39 ± 4.06 µg/mL, respectively, against MDA-MB-231 breast cancer cells (Figure 8A). After 72 h of incubation, the IC_50_ of the optimized PPN-LPHNPs and free PPN was observed to be 15.36 ± 2.34 µg/mL and 26.54 ± 2.79 µg/mL, respectively (Figure 8B). On the other hand, after an incubation period of 48 h, the optimized PPN-LPHNPs and free PPN was observed to be 21.32 ± 2.17 µg/mL and 37.42 ± 3.25 µg/mL, respectively, against MCF-7 cells (Figure 8C). After 72 h of incubation, the IC_50_ of the optimized PPN-LPHNPs and free PPN was observed to be 9.46 ± 2.18 µg/mL and 23.38 ± 3.12 µg/mL, respectively (Figure 8D). These findings confirmed that the LPHNPs exhibited very effective killing of both cancer cells when compared with the free PPN. The enhanced anti-breast cancer efficacy of PPN-LPHNPs was ascribed to the small PS (<175 nm) as well as controlled release of PPN [38].

### 3.7. Ex Vivo PPN Permeation Study

**Figure 9:**
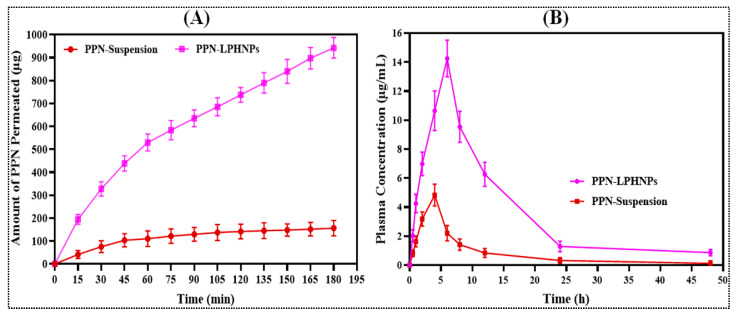
Image illustrating (**A**) comparative intestinal permeation profiles of PPN suspension and PPN-LPHNPs, (**B**) comparative plasma concentration vs. time profiles of PPN suspension and PPN-LPHNPs.

To evaluate the potential of PPN permeation encapsulated in the LPHNPs across the small intestine, a comparative ex vivo permeation study for PPN-LPHNPs and PPN suspension was conducted with the small intestine of albino Wistar rats, and the findings were compared to analyze the results. In this study, the intestinal permeation of PPN-LPHNPs and PPN suspension was quantified as 942.43 ± 45.39 μg/cm^2^ and 156.38 ± 33.47 μg/cm^2^, respectively, as depicted in Figure 9A. Therefore, PPN-LPHNPs exhibited 6.02-times higher (*p* < 0.05) intestinal permeation in comparison to the PPN suspension. Furthermore, a significant (*p* < 0.05) improvement in the APC for PPN-LPHNPs was also achieved. PPN-LPHNPs exhibited the APC of 2.1 × 10^−3^ cm^2^/h while the PPN suspension showed an APC of 1.89 × 10^−4^ cm^2^/h. Furthermore, the ER of PPN-LPHNPs was found to be more than nine times higher when compared with the PPN suspension. This dramatic improvement in the intestinal permeability of PPN-LPHNPs was ascribed to the small-sized particles (<175 nm) that represent a significantly greater surface area for intestinal absorption. Another major factor behind greater intestinal absorption is the positive ZP of PPN-LPHNPs. Positively charged PPN-LPHNPs significantly interact with the intestinal mucosa and open tight junctions, thereby significantly improving the permeation of encapsulated drugs [50].

### 3.8. Pharmacokinetic Study

To investigate the potential of LPHNPs in the enhancement of the oral bioavailability of encapsulated PPN, a comparative pharmacokinetic study for PPN-LPHNPs and PPN suspension was conducted in albino Wistar rats after a single-dose administration; the findings were compared to analyze the results. Different pharmacokinetic parameters observed for PPN-LPHNPs as well as conventional PPN suspension are summarized in Table 6, and the plasma profiles of PPN-LPHNPs and PPN suspension after oral administration of a single dosage equivalent to 20 mg/kg to the albino Wistar rats are represented in Figure 9B. As per the results, PPN-LPHNPs revealed significantly higher plasma PPN concentration compared to PPN suspension at each time point. PPN-LPHNPs exhibited an area under curve (${\mathrm{AUC}}_{0\to 24}$) and maximum plasma concentration ($C_{\max}$) of 176.82 µh/mL and 14.25 µg/mL, respectively. PPN suspension exhibited ${\mathrm{AUC}}_{0\to 24}$ and $C_{\max}$ of 38.842 µh/mL and 4.83 µg/mL, respectively. Therefore, PPN-LPHNPs revealed 4.552- and 2.95-times improved (*p* < 0.05) relative oral bioavailability and $C_{\max}$ in comparison to the PPN suspension. Furthermore, the mean resident time (MRT), time to reach maximum concentration ($T_{\max}$), plasma half-life ($t_{1/2}$), and elimination rate constant ($K_{\mathrm{el}}$) for PPN-LPHNPs was calculated to be 12.267 h, 6 h, 15.445 h, and 0.044 h^−1^, respectively. PPN suspension exhibited an MRT, $T_{\max}$, $t_{1/2}$, and $K_{\mathrm{el}}$ of 10.363 h, 4 h, 10.769 h, and 0.064 h^−1^, respectively. Therefore, the optimized PPN-LPHNPs revealed much higher (i) residence time in the systemic circulation, (ii) biological half-life, and slow clearance from the systemic circulation compared to the PPN suspension. The poor biological concentration of PP suspension was achieved due to poor absorption from the small intestine, which corresponds to the very poor solubility of PPN in the intestinal milieu. On the other hand, the absorption and biological concentration after oral administration of the optimized PPN-LPHNPs was ascribed to the higher surface area for intestinal absorption, the presence of PPN in the amorphous state in the matrix of LPHNPs, and the controlled release of PPN from the unique hybrid matrix of LPHNPs [51,52]. Furthermore, the mucoadhesive characteristic of the optimized PPN-LPHNPs is another major factor behind the higher oral absorption of PPN. The mucoadhesiveness of PPN-LPHNPs helps them to adhere to the mucous membrane and increases the residence time in GIT significantly [53,54].

## 4. Conclusions

In the present investigation, novel mucoadhesive PPN-LPHNPs were developed and optimized by using 3^3^-BBD for improved oral bioavailability. PPN-LPHNPs revealed excellent gastrointestinal as well as colloidal stability and sustained release profiles up to 24 h. The cell culture experiment revealed significantly improved cytotoxicity of PPN-LPHNPs against both MDA-MB-231 as well as MCF-7 cells. In addition, the optimized LPHNPs exhibited more than six-times higher intestinal permeation compared to conventional suspension. Furthermore, after a single-dose administration in albino Wistar rats, the optimized PPN-LPHNPs exhibited more than 4.5-times higher relative oral bioavailability compared to the conventional PPN suspension. Collectively, our investigation suggested that the LPHNPs can be a promising nanocarrier for improved oral delivery of PPN. Nevertheless, further studies including in vivo anti-breast cancer activity, biodistribution, and toxicological assessment in the animal model are required to validate in vitro results of the present investigation. 

## Figures and Tables

**Table 1:** Selected independent and dependent variables that were used to optimize PPN-LPHNPs by the 3^3^-BBD.

Factor	Levels Used, Actual (Coded Factor)		
Independent variables	Low (−1)	Medium (0)	High (+1)
A = Concentration of PL-90G (mg)	75	100	125
B = Concentration of CS (mg)	40	60	80
C = Concentration of P-188 (mg)	50	75	100
Dependent variables	Goal		
R_1_ = Particle size (PS; nm)	Minimize		
R_2_ = Polydispersity index (PDI)	Minimize		
R_3_ = Entrapment efficiency (EE; %)	Maximize		

**Table 2:** Composition of PPN-LPHNPs with their experimental value of respective responses.

Formulations	Independent Variables			Dependent Variables		
	A	B	C	R_1_	R_2_	R_3_
F1	75	60	100	131.47	0.146	66.89
F2	125	40	75	175.11	0.259	70.51
F3	125	60	100	152.47	0.275	72.73
F4	100	60	75	159.72	0.231	78.46
F5	75	60	50	145.21	0.215	59.94
F6	100	80	100	177.81	0.285	78.43
F7	100	40	100	132.71	0.187	62.44
F8	125	80	75	218.76	0.436	81.34
F9	125	60	50	213.84	0.375	76.55
F10	100	40	50	168.83	0.257	63.02
F11	75	40	75	127.47	0.203	57.98
F12	100	80	50	211.83	0.385	71.37
F13	75	80	75	170.13	0.226	72.74
F14	100	60	75	160.12	0.234	78.58
F15	100	60	75	158.94	0.229	77.89

**Table 3:** Statistical summary of the applied model of 3^3^-BBD on R_1_ (PS), R_2_ (PDI), and R_3_ (%EE).

Model	R^2^	Adjusted R^2^	Predicted R^2^	SD	Remark
Response (R_1_)					
Linear	0.9018	0.8751	0.8034	10.32	–
2F1	0.9495	0.9116	0.7883	8.68	–
Quadratic	0.9992	0.9976	0.9878	1.39	Suggested
Response (R_2_)					
Linear	0.8606	0.8225	0.7353	0.0334	–
2F1	0.9334	0.8834	0.7878	0.0270	–
Quadratic	0.9981	0.9948	0.9722	0.0057	Suggested
Response (R_3_)					
Linear	0.6967	0.6140	0.5351	4.71	–
2F1	0.7557	0.5725	0.4750	4.96	–
Quadratic	0.9974	0.9927	0.9631	0.6462	Suggested

**Table 4:** ANOVA data obtained after regression analysis and lack-of-fit tests for the best-fitting quadratic model for R_1_ (PS), R_2_ (PDI), and R_3_ (%EE).

Model	Source	PS	PDI	%EE
Regression analysis				
Quadratic	Sum of Squares	11,920.87	0.0876	802.50
	df	9	9	9
	Mean Square	1324.54	0.0097	89.17
	F- Value	681.38	297.92	213.55
	*p*-value, Prob > F	<0.0001	<0.0001	<0.0001
	Remark	Suggested, significant		
Lack of fit tests				
Quadratic	Sum of Squares	9.00	0.0002	1.82
	df	3	3	3
	Mean Square	3.00	0.0001	0.6053
	F- Value	8.33	7.93	4.45
	*p*-value, Prob > F	0.1091	0.1140	0.1888
	Remark	Suggested, not significant		

**Table 5:** Stability of PPN-LPHNPs in SGF (pH 1.2) and SIF (pH 6.8).

Parameters	SGF (pH 1.2)		SIF (pH 6.8)	
	Initial	Final	Initial	Final
PS (nm)	158.72 ± 5.27	174.26 ± 5.71	158.72 ± 5.27	169.67 ± 4.57
PDI	0.231 ± 0.01	0.253 ± 0.02	0.231 ± 0.01	0.235 ± 0.01
EE (%)	78.46 ± 3.34	74.37 ± 4.17	78.46 ± 3.34	72.63 ± 3.54
ZP (mV)	+24.31 ± 2.41	+21.07 ± 2.14	+24.31 ± 2.41	+22.72 ± 2.36

**Table 6:** Pharmacokinetic parameters of PPN-LPHNPs and PPN suspension after a single-dose administration in albino Wistar rats.

Parameters	PPN-Suspension	PPN-LPHNPs
$C_{\max}$ (µg/mL)	4.83	14.25 *
$T_{\max}$ (h)	4	6
${\mathrm{AUC}}_{0\to 24}$ (µh/mL)	38.842	176.82 *
${\mathrm{AUC}}_{0\to \infty }$ (µh/mL)	40.679	196.206 *
${\mathrm{AUMC}}_{0\to 24}$ (µh^2^/mL)	402.527	2169.082 *
${\mathrm{AUMC}}_{0\to \infty }$ (µh^2^/mL)	522.736	3531.615 *
MRT (h)	10.363	12.267
$t_{1/2}$ (h)	10.769	15.445
$K_{\mathrm{el}}$ (h^−1^)	0.064	0.044
$F_{\mathrm{rel}}$	–	4.55

* Denotes significantly (*p* < 0.05) different values of the optimized PPN-LPHNPs compared to PPN-suspension.

## Data Availability

The data presented in this study are available in manuscript.
